# Hepatitis C virus eradication improves immediate and delayed episodic memory in patients treated with interferon and ribavirin

**DOI:** 10.1186/s12876-017-0679-5

**Published:** 2017-11-25

**Authors:** Mary Ellen Dias Barbosa, Ana Luiza Zaninotto, Daniel Ferraz de Campos Mazo, Mario Guimarães Pessoa, Cláudia Pinto Marques Souza de Oliveira, Flair José Carrilho, Alberto Queiroz Farias

**Affiliations:** 1 Division of Psychology, Clinics Hospital, University of Sao Paulo School of Medicine, Avenida Dr. Eneas Carvalho de Aguiar, 255, São Paulo, 05403-900 Brazil; 2 Division of Clinical Gastroenterology and Hepatology, Clinics Hospital, Department of Gastroenterology, University of São Paulo School of Medicine, Avenida Dr. Eneas Carvalho de Aguiar, 255, sala 9159, São Paulo, 05403-900 Brazil; 3 Division of Gastroenterology, School of Medical Sciences, University of Campinas, Rua Carlos Chagas, 420, Campinas, 13083-878 Brazil; 4 Department of Gastroenterology, University of Sao Paulo School of Medicine, Av. Dr. Enéas de Carvalho Aguiar n° 255, São Paulo, 05403-000 Brazil

**Keywords:** Cognition, Memory, Attention, Neuropsychology, Hepatitis C, Depression

## Abstract

**Background:**

Chronic hepatitis C virus (HCV) infection is associated with impairment of cognitive function and mood disorders. Our aim was to evaluate the impact of sustained virological response (SVR) on cognitive function and mood disorders.

**Method:**

A prospective exploratory one arm study was conducted. Adult clinically compensated HVC patients were consecutively recruited before treatment with interferon and ribavirin for 24 to 48 weeks, according to HCV genotype. Clinical, neurocognitive and mood assessments using the PRIME-MD and BDI instruments were performed at baseline, right after half of the expected treatment has been reached and 6 months after the end of antiviral treatment. Exclusion criteria were the use of illicit psychotropic substances, mental confusion, hepatic encephalopathy, hepatocellular carcinoma, severe anemia, untreated hypothyroidism, Addison syndrome and major depression before treatment.

**Results:**

Thirty six patients were enrolled and 21 completed HCV treatment (*n* = 16 with SVR and *n* = 5 without). Regardless of the viral clearance at the end of treatment, there was a significant improvement in the immediate verbal episodic memory (*p* = 0.010), delayed verbal episodic memory (*p* = 0.007), selective attention (*p* < 0.001) and phonemic fluency (*p* = 0.043). Patients with SVR displayed significant improvement in immediate (*p* = 0.045) and delayed verbal episodic memory (*p* = 0.040) compared to baseline. The baseline frequency of depression was 9.5%, which rose to 52.4% during treatment, and returned to 9.5% 6 months after the end of treatment, without significant difference between patients with and without SVR. Depressive symptoms were observed in 19.1% before treatment, 62% during (*p* = 0.016) and 28.6% 6 months after the end of treatment (*p* = 0.719).

**Conclusions:**

Eradication of HCV infection improved cognitive performance but did not affect the frequency of depressive symptoms at least in the short range.

## Background

Hepatitis C virus (HCV) infection is a global public health problem. It is estimated that between 130 and 185 million people are infected worldwide [1]. Roughly 85% of patients infected go on to develop chronic hepatitis and 20 to 40% develop end-stage liver disease (i.e. cirrhosis) within 10 to 20 years [2]. In addition to liver disease, HCV is related to a series of extrahepatic manifestations, with a negative impact on the physical and mental health of infected individuals [3]. Patients with advanced cirrhosis commonly display hepatic encephalopathy, with characteristic neuropsychological, behavioral and neurological changes. However, in recent years a number of studies have established that up to a third of patients without cirrhosis also develop a neuropsychiatric syndrome characterized by impairment of cognitive function and symptoms related to depression [4–6]. These abnormalities were shown by neuropsychological tests, magnetic resonance spectroscopy and other neuroimaging modalities and various inventories of depression [4, 7, 8]. The main cognitive abnormalities include impairment of attention span, concentration and psychomotor skills. Depressive symptoms are found in roughly 40% of patients and reach a prevalence of 20% to 40% during antiviral treatment, particularly with interferon, with detrimental effects on both quality of life and compliance with treatment [4, 9].

Various lines of evidence suggest the cognitive dysfunction and depressive symptoms are related to the release of pro-inflammatory cytokines caused by infection of the central nervous system by HCV. HCV replicates itself in mononuclear peripheral blood cells and in bone marrow, which are precursors to the microglial cells in the brain. It is in this way HCV is introduced to the central nervous system via a “Trojan horse” mechanism [4, 10, 11]. However, no clear correlation between HCV viral load and cognitive impairment could be demonstrated [12]. Despite HCV infection itself within the brain causing local inflammation and neurocognitive disturbances, another hypothesis suggest these disturbances, including depression, could be in part a result of elevated systemic cytokine levels due to chronic HCV activation of the immune system [13–15]. Putative inflammatory cytokines are interleukin-1, 4, and 6, tumor necrosis factor-α and interferon-α, that can cross the blood-brain barrier and impact brain functioning [14, 15].

Despite various rigorous studies having demonstrated that HCV infection is associated with a decline in neurocognitive function and depressive symptoms, data are scarce as to whether or not these changes are irreversible in patients who have eliminated HCV after successful treatment [4, 6, 8]. Therefore, we conducted the present study with the aim of evaluating the impact of sustained virological response (SVR), i.e. viral clearance, in improving neurocognitive function and mood disorders in patients infected with HCV.

## Methods

### Subjects

The participants were selected from a teaching hospital and national centre of reference for Hepatology (Hepatology Outpatient Center at the Department of Gastroenterology, Clinics Hospital, University of São Paulo School of Medicine, Brazil) between 2011 and 2013. The inclusion criteria were: outpatients between the ages of 18 and 70, eligible for antiviral treatment of chronic HCV infection. The exclusion criteria were: use of psychotropic substances, mental confusion evaluated by the Mini-Mental State Examination with a score < 24 points [16], overt hepatic encephalopathy identified under West Haven criteria [17], minimal/subclinical hepatic encephalopathy shown by the Inhibitory Control Test [18], hepatocellular carcinoma, severe anemia, untreated hypothyroidism, Addison syndrome and major depression before treatment. Alcohol intake was assessed in all patients by the Alcohol Use Disorders Identification Test –AUDIT [19].

### Study design

A prospective exploratory one arm study was conducted in which the patients were assessed before the beginning, right after half of the expected treatment has been reached and 6 months after completion of antiviral therapy. All study participants underwent psychometric testing under the supervision of a psychologist and a neuropsychologist.

All patients were submitted to clinical assessment and blood tests for liver enzymes, hemoglobin, thyroid stimulating hormone, HCV genotype and viral load. HIV and hepatitis B co-infection were excluded using the appropriate serological tests. HCV-RNA was detected with real time polymerase chain reaction with kits purchased from Abbott Molecular (Illinois, USA). This assay achieves an inter-assay standard deviation of less than or equal to 0.25 log IU/mL of HCV RNA for samples containing HCV concentrations from 100 to 100 million IU/mL. The inter-assay SD at 5.97 and at 1.96 log IU/mL were of 0.04 and 0.09, respectively. Limits of detection were 12 to 100.000.000 IU/mL. Liver biopsy was carried out on all patients, with the exception of 3 (*n* = 2, hemophilia; *n* = 1 severe thrombocytopenia). The liver biopsies were carried out with a 14-G Tru-Cut™ needle (Medical Technology, Gainsville, FL, USA). Liver histology was examined by an experienced liver pathologist from the Department of Pathology of University of São Paulo School of Medicine. The classification score used for analysis of the biopsies was the METAVIR score: F0 - no fibrosis; F1 - portal fibrosis without septa; F2 - portal fibrosis and few septa extending into lobules; F3 - numerous septa extending to adjacent portal tracts or terminal hepatic venules and F4 - cirrhosis [20].

The antiviral therapy consisted of subcutaneous injections of interferon administered weekly and ribavirin administered daily and adjusted to the weight of the patient over a period of 24 weeks (genotypes 2 and 3) or for 48 weeks (genotypes 1 and 5), according to scientific recommendations of HCV treatment at the time the study was performed [21]. SVR was defined as a negative HCV RNA polymerase chain reaction assay 24 weeks after cessation of hepatitis C treatment. Patients without early virological response were defined as those with absence of at least 2 log reduction in HCV RNA at 12 weeks of treatment initiation.

### Neuropsychological assessment

#### Executive functions

##### Victoria Stroop test [22, 23]

Selective Attention and inhibition - Consisting of three cards: first (color identification), second (identification of colored words) and third (color interference). On each card, the subject is encouraged to give the name of the color of the ink as quickly as possible. The score is calculated by the time spent on each card.

##### Trail Making Test A and B (TMT form A and B) [22, 24, 25]

Sustained (TMT form A) and Alternated Attention (TMT form B). Consist of 25 circles distributed over a sheet of paper. In Form A, the circles are numbered 1–25, In Part B, the circles include both numbers (1–13) and letters (A – L); as in Part A, the patient draws lines to connect the circles in an ascending pattern, but with the added task of alternating between the numbers and letters (i.e., 1-A-2-B-3-C, etc.). The test is stopped if the patient has not completed both parts after 5 min have elapsed. The score is calculated by the time spent on each trial.

##### Phonemic Fluency – (COWAT FAS) [22]

Assessed by asking the patient to say as many words as possible that begin with the letters F, then the letter A, then the letter S over the course of 1 min. Proper nouns, such as of people or places, are not permitted, nor are conjugations of verbs. The score is given as the sum of the correctly spoken words.

##### Semantic fluency [22]

Assessed by asking the patient to recite the names of as many animals as possible in 1 min. For the scoring, words belonging to a larger category are not considered if the patient has already used words from the subcategory. Each word is afforded a score of 1.

#### Episodic verbal memory and learning

##### Hopkins Verbal Learning Test - Revised- HVLT-R [26]

Verbal Learning and Long-term episodic memory. After the examiner reads the list of 12 words, the patient is asked to repeat as many words as possible, in any order (immediate recall). This process is repeated twice more and after 25 min a delayed recall is sought. Recognition is also required. Each right word scores 1 point.

#### Working memory

##### Forward and backward digit span [27]

To assess the work memory, increasing sequences of digits are orally presented at a rate of one per second. At the end of each sequence, the subjects must repeat them in direct order (forward) and inverse order (backward). The score represents the number of digits counted in the largest correctly repeated sequence forward and backward.

#### Estimated Intelligence Quotient (IQ)

##### Vocabulary and reasoning matrices

Subtest of Wechsler Adult Intelligent Scale (WAIS III) [27]. In the vocabulary assessment, 33 words are orally presented and the patient should define or explain their meanings. Six consecutive words incorrectly defined mark the end of the test. Each word receives a score of 0, 1 or 2. The subtest Reasoning Matrix consists of a logical sequence of figures presented incompletely and with a progressive difficulty which the patient chooses between six others, to complete a logical sequence. Eight consecutive incorrect responses end the test. The sum of the subtest scores gives an estimate of intellectual quotient.

#### Evaluation of depression and depressive symptoms

##### Primary Care Evaluation of Mental Disorders – PRIME – MD [28, 29]

The module of major depression of the structured interview PRIME-MD, which is based on the diagnostic criteria of DSM-IV [30] was used because it is considered the gold standard of psychopathological disturbance assessment.

##### Beck Depression Inventory – BDI [31]

The self-evaluation inventory numerically demonstrates descriptive items, attitudes and depressive symptoms to assess different conditions found in the general population. It was used in this study for the evaluation of depressive symptoms. The cutoff scores recommended for evaluation (by the non-professional population) were: 0–9 absence of depressive symptoms or minimal symptoms; 10–16 mild symptoms; 17–29 moderate symptoms; 30–63 severe symptoms.

### Statistical analysis

Fisher exact test was used for comparison of patients with and without SVR. Student t and Mann –Whitney tests were used for continuous variables. Results were expressed as mean, standard deviation, percentage and Z-score. *Z-*score was used only for the neuropsychological tests and was obtained via the conversion of the points of the neuropsychological tests through the mean and standard deviation from the normative population. Values of z-score ≤ **−**1.50 were considered impairment. Covariate analysis was used to assess years of education and time as potential confounders. All data were analyzed as complete protocol. The R program version 3.12.1 (R Core Team, Auckland, New Zealand, 2014) was used for calculations. *P* value < 0.05 was considered statistically significant. A statistical review of the study was performed by a biomedical statistician.

## Results

### Patients

**Fig. 1:**
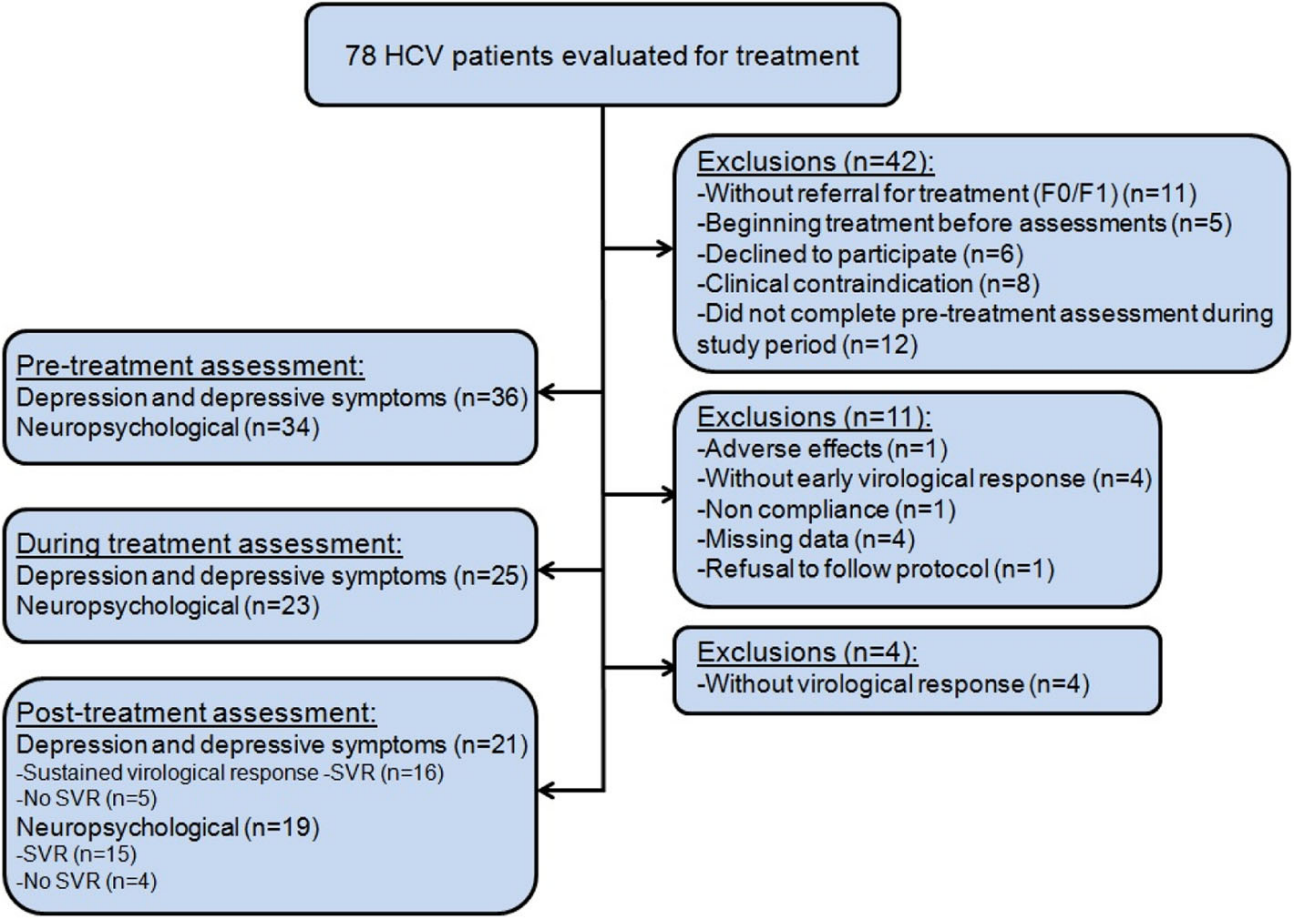
Study flow chart

Seventy eight patients were consecutively recruited at the Hepatology Outpatient Center at the Department of Gastroenterology. Forty two patients of these were excluded (see Fig. 1). The remaining 36 comprise the present series, as shown in Fig. 1.

Baseline characteristics of included patients are provided in Table 1. Most of the patients were female in the middle age, with HCV genotype 1 and absence of advanced liver fibrosis. During the antiviral treatment, patients maintained appropriate levels of hemoglobin. Twenty one patients completed antiviral treatment. None of the participants displayed problematic alcohol intake, mental confusion or hepatic encephalopathy at inclusion, during and after treatment. Twenty three (63.9%) patients had other illnesses or associated conditions, i.e. arterial hypertension, *n* = 15 (65.2%); diabetes mellitus, *n* = 5 (21.7%); arthrosis/osteoporosis, *n* = 5 (21.7%); cardiopathy, *n* = 4 (17.4%); well-controlled hypothyroidism, *n* = 3 (13%); hemophilia, *n* = 2 (8.7%); nephrotic syndrome, *n* = 2 (8.7%) and miscellaneous, *n* = 5 (21.7%).

**Table 1:** Baseline characteristics

Characteristics	Patients (*n* = 36)
Age in years ± SD (range)	49.5 ± 12.04 (29–69)
Female (%)	20 (55.6)
Education level (%)	
University	7 (19.4)
High school	12 (33.3)
Primary school	17 (19.4)
Occupation (%)	
Employed	23 (63.9)
Unemployed	7 (19.4)
Retired	6 (16.7)
HCV genotype (%)	
Genotype 1	27 (75.0)
Genotype 3	7 (19.4)
Other genotypes	2 (5.6)
Histological stage (%)	
F1/F2	26 (72.2)
F3	4 (19.0)
F4	6 (8.8)

### Neuropsychological assessment

Nineteen patients had the three neuropsychological assessments (before, during and after HCV treatment). Comparing before and during treatment (right after half of the expected treatment has been reached, at week 12 or 24), baseline z-score for selective attention was −2.10 ± 1.28 and improved to −1.04 ± 0.93 during antiviral therapy (*p* = 0.004). No improvement in other neurocognitive domains was observed during interferon-based therapy, as shown in Table 2.

**Table 2:** Neuropsychological domains in patients before and during antiviral treatment

Domain	Baseline (*n* = 19)	During treatment (*n* = 19)	*p*-value
	M ± SD*	M ± SD*	
Immediate episodic memory	−1.50 ± 1.05	−1.34 ± 1.01	0.758
Delay episodic memory	−1.36 ± 1.12	−1.20 ± 1.31	0.433
Memory recognition	−0.19 ± 1.11	0.61 ± 1.24	0.167
Working memory	0.12 ± 0.65	0.26 ± 0.81	0.576
Focused attention	−0.86 ± 1.49	−0.80 ± 1.35	0.850
Divided attention	−1.51 ± 1.47	−0.85 ± 1.19	0.433
Selective attention	−2.10 ± 1.28	−1.04 ± 0.93	0.004**
Phonemic verbal fluency	−0.73 ± 1.13	−0.67 ± 0.95	0.811
Semantic verbal fluency	−0.39 ± 1.15	−0.85 ± 1.09	0.136
Dominant hand coordination	−1.62 ± 1.27	−1.35 ± 1.37	0.344
Non dominant hand coordination	−1.58 ± 1.23	−1.70 ± 1.17	0.836

*M* mean, *SD* standard deviation

*z score; ** *p* < 0.05

Regardless of the viral clearance, there was significant improvement in the domains of immediate verbal episodic memory, delayed verbal episodic memory, selective attention and phonemic verbal fluency (*p* < 0.05) after the end of treatment (Table 3).

**Table 3:** Neuropsychological domains in patients before and after antiviral treatment, regardless of the viral clearance

Domain	Baseline (*n* = 19)	6 months after treatment (*n* = 19)	*p*-value
	M ± SD*	M ± SD*	
Immediate episodic memory	−1.50 ± 1.05	−0.74 ± 0.92	0.010**
Delay episodic memory	−1.36 ± 1.12	−0.79 ± 1.22	0.007**
Memory recognition	−0.19 ± 1.11	0.13 ± 1.18	0.293
Working memory	0.12 ± 0.65	0.15 ± 0.51	0.670
Focused attention	−0.86 ± 1.49	−0.65 ± 1.51	0.421
Divided attention	−1.51 ± 1.47	−0.50 ± 1.22	0.142
Selective attention	−2.10 ± 1.28	−0.20 ± 0.97	0.000**
Phonemic verbal fluency	−0.73 ± 1.13	−0.34 ± 1.08	0.043**
Semantic verbal fluency	−0.39 ± 1.15	−0.53 ± 1.04	0.705
Dominant hand coordination	−1.62 ± 1.27	−1.24 ± 1.57	0.258
Non dominant hand coordination	−1.58 ± 1.23	−1.18 ± 1.79	0.298

*M* mean, *SD* standard deviation

*z score; ** *p* < 0.05

Of the 19 patients who completed HCV treatment, 15 patients had SVR. This subgroup of patients with SVR presented significant improvement in the domains of immediate verbal episodic memory (*p* = 0.045) and delayed verbal episodic memory (*p* = 0.040) when compared with the group without SVR, as shown in Table 4.

**Table 4:** Neuropsychological domains according to the virological response to treatment

Domain	With SVR (*n* = 15)	Without SVR (*n* = 4)	*p*-value
	M ± SD*	M ± SD*	
Immediate episodic memory	−0.50 ± 0.80	−1.65 ± 0.84	0.045**
Delay episodic memory	−0.49 ± 1.12	−1.90 ± 0.99	0.040**
Memory recognition	0.38 ± 0.81	−0.80 ± 1.94	0.736
Working memory	0.17 ± 0.54	0.08 ± 0.42	0.760
Focused attention	−0.35 ± 1.39	−1.80 ± 1.58	0.064
Divided attention	−0.53 ± 1.31	−0.30 ± 0.28	1.000
Selective attention	−0.11 ± 0.68	−0.5 ± 1.80	1.000
Phonemic verbal fluency	−0.14 ± 1.05	−1.09 ± 0.93	0.160
Semantic verbal fluency	−0.40 ± 1,08	−1.02 ± 0.83	0.208
Dominant hand coordination	−1.02 ± 1.61	−2.07 ± 1.26	0.228
Non dominant hand coordination	−1.15 ± 1.92	−1.26 ± 1.44	0.920

*M* mean, *SD* standard deviation, *SVR* sustained virological response

*z score; ** *p* < 0.05

### Evaluation of depression and depressive symptoms

Twenty one patients had the three depression and depressive symptoms assessments (before, during and after HCV treatment). The baseline frequency of major depression was 9.5% (2 patients), rising to 52.4% (11 patients) during treatment (*p* = 0.012) and returning to 9.5% 6 months after the end of treatment, with no difference between patients with and without SVR (*p* = 0.429). In a similar way, depressive symptoms were observed in 19.1% before treatment, reaching 62% during (*p* = 0.016) and reducing to 28.6% after the end of treatment (*p* = 0.719). There were no differences regarding depressive symptoms according to virological response (*p* = 0.597).

The covariate analysis showed the impact of higher years of schooling as a predictor of better results at neuropsychological tests (*p* < 0.01).

## Discussion

In this study, the neuropsychological performance and the frequency of major depression and depressive symptoms of patients infected with HCV were compared before the beginning of treatment, right after half of the expected treatment has been reached and 6 months after the end of treatment. We observed that patients that attained viral clearance (SVR) improved significantly in the neurocognitive domains of immediate and delayed verbal episodic memory compared to patients without.

Since the seminal report by Forton et al. [32] describing an elevated choline/creatinine ratio by proton magnetic resonance spectroscopy in the frontal white matter and basal ganglia of HCV-infected patients, several studies have suggested that the brain may be affected in chronic HCV infection. Subsequent reports have documented that up to one third of HCV infected patients may have neuropsychological impairment [7, 11, 12, 33–35]. These changes are seen even in the absence of cirrhosis, decompensated liver disease, hepatic encephalopathy or previous history of abuse of psychoactive substances, suggesting an effect of the viral infection on the central nervous system. HCV locally induced inflammation and the passage of systemic pro-inflammatory cytokine through the blood-brain barrier could also impair neurologic functioning [14, 15]. The neurocognitive disturbances may be documented long before the beginning of interferon-based antiviral therapy, which is a well-known cause of neurocognitive, mood and psychiatric dysfunction.

Most studies were based on the comparison of the brain of infected versus non-infected patients by magnetic resonance imaging and functional single photon emission tomography [7, 12, 33–35]. Despite the high degree of heterogeneity of these studies, it is indisputable that chronic HCV infection is associated with impairment of attention, executive function, verbal abilities and memory. In this regard, it is noteworthy that few studies used a structured and validated neuropsychological battery of tests for diagnosing this neurocognitive dysfunction.

In contrast with a number of papers that show neuropsychological impairment in HCV infected patients, few data are available describing the potential reversibility of the cognitive disturbances after successful antiviral treatment. Byrnes et al. [36] performed ^1^H magnetic resonance spectroscopy and a battery of neuropsychological tests before, during and after antiviral treatment with interferon and ribavirin in 15 HCV-infected patients. They concluded that HCV eradication had a beneficial effect on cerebral metabolism and improved verbal learning and visual and spatial memory. However, the sample size was limited and post-treatment assessment was carried out 12 weeks after the discontinuation of interferon, not enough to fulfill the definition of SVR. In a recent paper, Kraus et al. [37] performed a multicenter study including 168 HCV patients receiving antiviral therapy with interferon and ribavirin. Twelve months after the termination of antiviral treatment, patients with SVR had significant improvement in 3 out 5 domains (vigilance, shared attention: optical task and working memory). Our findings confirm previous few observations that HCV-associated neurocognitive impairment may be reversible after viral eradication. However, our study differs from the paper of Byrnes et al. [36] because we demonstrated that neurocognitive improvement persist long after the withdrawal of the therapy in patients with SVR, as defined by the inability to detect HCV RNA 24 weeks after completion of treatment. Despite the improvement in HVLT immediate and delay recall from baseline in those attaining SVR in our study, it is noteworthy that subjects are still performing in the impaired range. Persistent neurocognitive impairment in patients treated with interferon and ribavirin was described by Cattie et al., specifically within the domains of working memory, learning, and executive functions, even after HCV viral load undetectability [38]. Our study has also some difference with the paper of Kraus et al. [37]. We used a stringent protocol that included clinical assessment, exclusion of potential confounding variables such as unrelated conditions (severe anemia, fatigue, untreated hypothyroidism, and dementia), substance abuse and heavy alcohol intake. Cognitive function disturbances were assessed by a more comprehensive battery of neuropsychological tests, which included 11 domains of interest in HCV-infected patients. Mood was assessed with two different instruments. Furthermore, socio-demographical variables which could influence the performance of the patients were taken into account.

Our study observed that depression and depressive symptoms are common in untreated hepatitis C patients and they increase significantly during interferon based treatment. However, SVR did not lead to a significant decline in the frequency of these symptoms. It is worth restating that the majority of patients displayed a related condition. In fact, the illness, regardless of its cause, can influence the presence of mood disorders. It is possible that the frequency of major depression and related symptoms observed before antiviral treatment reflect, at least in part, the clinical background of the patients. It is well documented in the literature that the frequency of depression and depressive symptoms increases over the course of interferon based treatment [39–41], but returns to baseline levels after the cessation of treatment, indicating the reversibility of these symptoms, observations confirmed by this study. Recently Huckans et al. also reported increased symptoms of depression during interferon therapy in a cohort of 33 HCV infected patients, which decreased or remitted following treatment discontinuation [42]. Eccles et al. have demonstrated that hypothalamic-pituitary-adrenal axis hyperactivity prior to interferon based therapy evaluated through measurement of the waking salivary cortisol response was associated to depression during treatment [43]. Whale et al. showed recently that younger age, previous history of major depression disorder, higher baseline psychomotor retardation and somatic symptoms item scores using the Hamilton Depression Rating Scale and HCV genotype 2 were implicated in depression during IFN treatment [44]. In the present study we excluded patients with major depression diagnosis at baseline and we could not find predictive factors for depression during IFN treatment, maybe due to small sample evaluated. The exogenous administration of IFN in addition to the HCV chronic activation of the immune and elevated systemic cytokine levels could induce sickness behavior and depression [13]. Interestingly, depression and / or the severity of depressive symptoms related to IFN treatment seem higher in HCV mono-infected patients when compared to HIV/HCV co-infected patients and in those with chronic hepatitis B [45, 46]. It is notable that hemolytic anemia induced by ribavirin is common during antiviral treatment, contributing to the somatic effects of depression. An American study with 32 patients concluded that interferon exacerbates somatic depressive symptoms [39]. However, the major limitation of this study pertains to the fact that the data were not controlled for anemia, and only US war veterans were included in the study, which is possibly not a representative sample of the population infected with HCV. Our data were rigorously controlled for hemoglobin levels throughout the course of treatment.

The frequent impairment of attention, concentration and psychomotor skills suggests a relation with the fronto-cortical systems, which would be affected by chronic HCV infection. Chronic HCV infection, independently of viral load genotype, triggers activation of inflammatory cytokines and immune response. This response is directed towards the 5-HT receptor, which plays an important role in the pathophysiology of depression and produces changes in cognitive function and mood [4, 32, 33]. Indeed, major depression may be associated with cognitive impairments, such as executive functions and memory alterations secondary to attention deficit [47]. In our study, however, despite an increase in depression and depressive symptoms during treatment, patients experienced improvement in the domains of immediate verbal episodic memory, delayed verbal episodic memory, selective attention and phonemic verbal fluency. Besides this, depression and depressive symptoms frequencies returned to baseline while immediate verbal episodic memory and delayed verbal episodic memory improved in those attaining SVR.

HCV therapy has experienced a revolution in recent years, with interferon free regimens directed at specific steps of viral replication (direct acting antivirals, i.e., DAA), leading to highly effective, shorter and safer treatments. To date, there are limited data regarding the impact of DAA induced viral clearance on cognition and depressive symptoms. Kleefeld et al. reported in an HCV / HIV population 12 patients who achieved viral clearance with DAA [48]. Improvement in the domains visual memory, attention, processing speed and executive functioning were reported, however, the limited number of patients and practice effects may hamper firm conclusions. Another study with sofosbuvir and lepipasvir associated HCV viral suppression with normalization of cerebral N-acetyl aspartate evaluated by magnetic resonance spectroscopy [49].

A limitation of our study is the lack of an untreated HCV control group to rule out some confounders, such as practice effect on the neurocognitive domains tested. Regarding the Trail Making Test A and B, we used it exclusively to evaluate the domain of sustained and alternated attention, but additional information about processing speed components could have been obtained. Another relative weakness of our study is the small number of patients, in particular those non-responders at the end of the follow-up.

## Conclusions

In summary, we demonstrated that patients which attained HCV eradication had significant improvement in immediate and delayed episodic memory. However, the frequency of depressive symptoms and depression did not decrease. The added benefit of the improvement of neurocognitive impairment after the HCV clearance with interferon-based therapy has clinical implications and the potential of making the cognitive function a valid outcome of treatment. Future studies will need to address the correlation of the performance on the battery of neurocognitive tests with depressive symptoms, quality of life or other patient reported outcomes. From the same perspective, further studies assessing the effect of the new DAA on the cognitive function are warranted.

## Abbreviations

AUDIT: Alcohol Use Disorders Identification Test
BDI: Beck depression inventory
COWAT-FAS: Controlled Oral Word Association Test- Phonemic Fluency
DAA: Directing acting antivirals
HCV: Cronic hepatitis C virus
HIV: Human immunodeficiency virus
HVLT-R: Hopkins verbal learning – revised
IQ: Intelligence quotient
PRIME-MD: Primary care evaluation of mental disorders
RNA: Ribonucleic acid
SVR: Sustained virological response
TMT A and B: Trail making test A and B
WAIS III: Wechsler adult intelligent scale
